# Digestion

**Published:** 1890-04

**Authors:** 


### DIGESTION.

Digestion will not begin till the temperature of the food has been raised by the heat of the stomach to ninety-eight degrees. Hence the more heat that can be imparted to it by slow mastication, the better. The precipitation of a large quantity of cold food into the stomach by fast eating may, and often does, cause discomfort and indigestion, and every occasion of this kind results in a measurable injury to the digestive functions. Ice water drank with cold food of course increases the mischief. Hot drinks—hot water, weak tea, coffee, chocolate, etc., will, on the contrary, help to prevent it. But eat slowly, any way.

